# Nrf2 Plays an Essential Role in Long-Term Brain Damage and Neuroprotection of Korean Red Ginseng in a Permanent Cerebral Ischemia Model

**DOI:** 10.3390/antiox8080273

**Published:** 2019-08-03

**Authors:** Lei Liu, Marie G. Kelly, Erika L. Wierzbicki, Iana C. Escober-Nario, Mary K. Vollmer, Sylvain Doré

**Affiliations:** 1 Department of Anesthesiology, Center for Translational Research in Neurodegenerative Disease and McKnight Brain Institute, University of Florida, Gainesville, FL 32610, USA; 2 Departments of Neurology, Psychiatry, Pharmaceutics, and Neuroscience, University of Florida, Gainesville, FL 32610, USA

**Keywords:** aquaporin 4, astrocyte, MCAO, microglia, neuroprotection, panax ginseng, reactive gliosis, recovery, stroke

## Abstract

Cerebral ischemia is a devastating disease with a high incidence of death and disability; however, effective therapeutics remain limited. The transcriptional factor Nrf2 has been shown to play a pivotal role in the endogenous defense against brain oxidative stress and inflammation and therefore represents a promising target for stroke intervention. However, the long-term effects of Nrf2 and the standardized Korean red ginseng (ginseng), a potent Nrf2 natural inducer, on permanent cerebral ischemic damage have not yet been reported. Wildtype (WT) and Nrf2^−/−^ adult mice were pretreated with either vehicle or ginseng and were subjected to permanent distal middle cerebral artery occlusion (pdMCAO). The infarct volume, the reactive astrocytes and microglia, and the water regulatory protein aquaporin 4 (AQP4) were examined at 28 days after stroke. When compared with the WT matched controls, the Nrf2 disruption significantly enlarged the infarct volume (40.4 ± 10.1%) and exacerbated the progression of reactive gliosis and AQP4 protein levels after pdMCAO. In contrast, ginseng significantly reduced the infarct volume and attenuated the reactive gliosis and AQP4 in the ischemic WT mice (47.3 ± 6.9%), but not in the Nrf2^−/−^ mice (25.5 ± 5.6%). In conclusion, Nrf2 plays an important role in the long-term recovery of permanent cerebral ischemic damage and the neuroprotection of ginseng.

## 1. Introduction

Cerebral ischemia is a devastating disease with a high incidence of mortality and disability [1]. It is caused by permanent or transient occlusion of blood vessels and their branches, which eventually leads to brain infarction. The final recovery in infarct size and neurological deficit is dependent on multiple factors, such as the duration of ischemia, brain location, lesion size, and ischemia-evoked endogenous protection. A better understating of the role of the key molecules involved in the pathophysiological consequences is highly important in the stroke field [1,2,3,4,5]. The cerebral ischemia mouse model of permanent distal middle cerebral artery occlusion (pdMCAO) provides a highly reproducible and well-standardized cortical lesion with a minimal mortality rate and closely mimics ischemic stroke in humans; therefore, it has been widely used in preclinical and translational research, allowing us to investigate long-term outcomes after stroke [6,7,8].

Increasing evidence indicates that the transcriptional factor Nrf2 plays a vital role in the endogenous defense against brain oxidative stress and inflammation, two crucial mechanisms of cerebral ischemic events [5,9]. Therefore, Nrf2 serves as a promising target for stroke intervention [5]. Recent advances have shed substantial light on the Nrf2 mechanism in stroke; however, our understanding of the Nrf2 function and targeted therapeutics in stroke remain limited [5]. The functional importance of Nrf2 has been reported in several pathological conditions, most of which came from studies with Nrf2^−/−^ mice [5,10,11]. Our group has been focused on Nrf2 and its targeted neuroprotectants for years [12,13,14,15,16,17,18]. The natural potential Nrf2-inducer ginseng, extracted from the root of *Panax ginseng* C.A. Meyer, has been widely used in East Asia for thousands of years, exhibiting potent anti-inflammatory and antioxidative properties [19,20]. Our previous study revealed the critical role of Nrf2 in acute cerebral ischemia and the efficacy of standardized Korean red ginseng extracts (ginseng); however, their long-term effects on ischemic brain damage after pdMCAO are unclear [12,18].

Reactive gliosis in astrocytes and microglia refers to the glial response to various central nervous system injuries with biochemical, morphological, and functional alterations, which play an essential role in stroke outcome and recovery [21,22]. Astrocytes play a critical role in attracting and restricting brain injury and show long-lasting benefits in stroke treatment [23,24]. Microglia, the primary cell type of neuroinflammation, are activated rapidly in response to ischemic insults in the peri-infarct and infarct core [25,26,27]. The astrocytic water channel aquaporin 4 (AQP4), the main water channel in the brain, is a key regulator of the formation and progression of edema associated with ischemia [12,18]. In addition, it was reported that the Nrf2 downstream target genes are enriched in glial cells, implying their potential close functional link with Nrf2 regulation [5,28,29].

In this study, by using the pdMCAO stroke model and Nrf2^−/−^ mice, we aimed to examine the effects of Nrf2 and ginseng on long-term cerebral ischemic damage. With histology and immunohistochemistry staining, the infarct volume, the reactive gliosis in astrocytes and microglia, and the AQP4 at 28 days after pdMCAO were examined. In addition, the contribution of Nrf2 in the potential neuroprotection of ginseng were analyzed by contrasting the measurements between post-pdMCAO wildtype (WT) and Nrf2^−/−^ mice. The insights into the link between Nrf2 and long-term ischemic brain injury will enhance our understanding of the pathogenesis of cerebral ischemia and the neuroprotective potential of ginseng. 

## 2. Materials and methods

### 2.1. Animals

The WT and the Nrf2^−/−^ mice had a C57BL/6 genetic background, as previously described [12,15,18,30]. Male mice 10 to 18 weeks old were used for this study. Mice were housed in a 12:12 reversed light/dark cycle with ad libitum access to water and food. All animal protocols were approved by the Institutional Animal Care and Use Committee of the University of Florida (IACUC approval #201605020) and were conducted according to the National Institute of Health guidelines. All animals were randomly distributed to groups. All efforts were made to reduce the animal number and to minimize animal suffering. The experimenters and analyzers were blinded to the genotype and treatment of mice.

### 2.2. Permanent Distal Cerebral Ischemia Mouse Model

The permanent ischemic stroke injury was produced by pdMCAO [12,31]. Briefly, mice were anesthetized by the inhalation of 5% isoflurane in air and were maintained under anesthesia with 2% isoflurane during surgery. Artificial tear ointment for protection and lubrication was applied to the eyes of each mouse. After a 0.8–1.0 cm incision was made between the right eye and ear, the temporal muscles were cautiously separated outward to visualize the bone. During a craniotomy, a piece of 2-mm^2^ temporal bone above the middle cerebral artery (MCA) was gently removed. The distal MCA was occluded permanently by electrocauterization (Bovie Medical Corp., Clearwater, FL, USA). After 30 s, the occlusion was checked again, and if necessary the coagulation was repeated. The sham-operated (sham) mice underwent an identical surgical procedure, except for the occlusion of pdMCA. During the surgery, a thermostat-controlled heating pad was used for maintaining the body temperature at 37 °C. After the surgery, the mice were moved into a standard temperature- and humidity-controlled environment for recovery.

### 2.3. Source of the Standardized Korean Red Ginseng Extract, Administration, and Animal Grouping

The standardized Korean red ginseng (ginseng), a water-soluble extract, was provided by Dr. Hocheol Kim and prepared as previously described [12]. Briefly, C57BL/6 WT and Nrf2^−/−^ mice were randomly divided into six groups: administered with ginseng (100 mg/kg/day; once daily; gavage) or vehicle (Veh, double-distilled water, 0.1 mL/kg) for 7 days, and then subject to pdMCAO surgery. The last administration was at 2 h before pdMCAO. At 28 days after pdMCAO, all mice were euthanized for different measurements: (1) WT: Veh + sham; (2) Nrf2^−/−^: Veh + sham; (3) WT: Veh + pdMCAO; (4) Nrf2^−/−^: Veh + pdMCAO; (5) WT: ginseng + pdMCAO; (6) Nrf2^−/−^: ginseng + pdMCAO. The animal number used for each analysis is indicated in the figure legends.

### 2.4. Evaluation of Cerebral Ischemic Infarct Volume

At day 28 after pdMCAO, mice were sacrificed for the following morphological analysis. The anesthetized mice were transcardially perfused with phosphate-buffered saline first and then with 4% paraformaldehyde. The mouse brains were harvested, post-fixed, and cryoprotected in 30% sucrose solution (w/v). Histology and immunohistochemistry staining were performed on 30-μm-thick frozen coronal sections [32].

The infarct volume was measured by Cresyl violet (CV) staining [12,32]. CV is widely used to visualize the Nissl bodies in the cells, which are the granular endoplasmic reticulum and ribosomes that occur in the soma and dendrites. The stain is dark blue, giving the cytoplasm a mottled appearance. These features were weakened or even not visible in damaged or dead neurons. The surrounding glial cells were stained at an extremely small size. Every tenth section throughout the infarct-containing region was stained and scanned (ScanScope CS; Aperio Technologies Inc., Vista, CA, USA) [33]. The border of the infarction area was identified with ImageScope Software (Aperio Technologies). The infarct sizes from all of the above sections were used for analyzing the total infarction volume.

### 2.5. Immunohistochemistry and Image Analysis

For immunohistochemistry, the sections were washed in phosphate buffered saline, blocked with 5% horse serum, and then incubated overnight with well-characterized primary antibodies for rabbit polyclonal glial fibrillary acidic protein (GFAP; 1:3000; catalog # Z0334; DAKO, Carpinteria, CA, USA), rabbit polyclonal ionized calcium-binding adapter protein 1 (Iba1; 1:5000; catalog #019-19741; Wako Bioproducts, Richmond, VA, USA), and rabbit polyclonal aquaporin 4 (AQP4; 1:3000; catalog #ab3594; EMD Millipore, Billerica, MA, USA). The tissues were thoroughly washed with phosphate buffered saline and were incubated with the appropriate secondary antibodies at room temperature for 2 h. The 3,3-diaminobenzidine chromogen solution (DAB substrate kit; Vector Laboratories, Burlingame, CA, USA) was used to visualize the expression levels for the above markers. The images for the staining were captured using ScanScope CS and analyzed using ImageScope Software (Aperio Technologies). 

To measure the immunoreactive intensities of the markers GFAP, AQP4, or Iba1, all of the images were set at the same threshold, and an indicated 250-μm square region of each section was selected for the quantification, as previously described [12]. For each staining, three consecutive sections per mouse (coronal levels around Bregma −0.46 mm) were analyzed, and the average value was determined per mouse. All analyses were performed by an experimenter blinded to the genotypes of the animals and treatments.

### 2.6. Statistical Analysis

All statistical analyses were performed using Prism version 5 (Graphpad Software, La Jolla, CA, USA). Multiple comparisons were analyzed using a two-way ANOVA, followed by a Bonferroni post hoc test. Data are reported as mean ± SEM. Statistical significance was set at *p* ≤ 0.05. The sample number for each measurement is stated in each figure legend.

## 3. Results

### 3.1. Loss of Nrf2 Exacerbated the Long-Term Cortical Lesion and Reduced Ginseng-Induced Neuroprotection after pdMCAO

**Figure 1:**
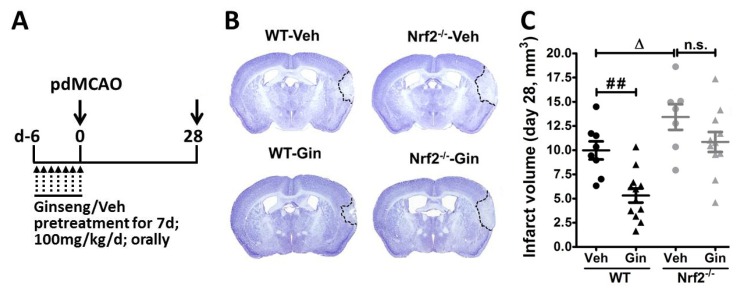
Ginseng promotes the long-term recovery of permanent cerebral ischemic damage in an Nrf2-dependent manner. (**A**) Experimental design. All mice received 7 days of oral administration of ginseng (Gin) or Vehicle (Veh; 100mg/kg/d; once daily), and were then subject to pdMCAO. The last administration was performed at 2 h before pdMCAO. (**B** and **C**) At 28 days after pdMCAO, the effect of ginseng on ischemic brain lesion was determined by Cresyl violet staining. In the vehicle-pretreated groups, the Nrf2^−/−^ mice displayed marked larger infarct volumes than the wildtype (WT) controls, supporting the critical role of Nrf2 in the long-term recovery of ischemic brain lesions. In contrast, the infarct volume was significantly reduced in ginseng-pretreated WT mice, but not Nrf2^−/−^ mice, suggesting potential Nrf2-dependent neuroprotection by ginseng. ^##^ *p* < 0.01, ^∆^ *p* < 0.05. WT-Veh: *n* = 7; WT-Gin: *n* = 12; Nrf2^−/−^-Veh: *n* = 7; Nrf2^−/−^-Gin: *n* = 11; pdMCAO: permanent distal middle cerebral artery occlusion; d: day; Gin: ginseng; Veh: vehicle; n.s.: no significance.

Previous studies from our group revealed that Nrf2 deficiency exacerbated acute brain damage and long-term functional deficits in a pdMCAO mouse model. In contrast, ginseng pretreatment conferred neuroprotection against such acute brain damage through Nrf2 mechanisms that eventually facilitated long-term functional recovery [12,18]. Thus, we sought to determine whether the loss of Nrf2 can impede the long-term recovery of pdMCAO-caused brain lesions and whether Nrf2 is essentially needed for the potential long-lasting neuroprotection of ginseng. Accordingly, we measured the infarct volume at 28 days after pdMCAO, using a widely used method of CV staining. The changes in brain lesion correlated with our previous finding on the long-term neurological deficits [12]. As shown in Figure 1, the infarct areas were predominantly in the cortex region. The Nrf2^−/−^ mice displayed larger infarct volume compared with WT controls at 28 days after pdMCAO (11.47 ± 0.94% vs. 14.92 ± 1.32%; *p* < 0.05), emphasizing the important beneficial role of Nrf2 in the recovery of cerebral ischemia. In contrast, the infarct volume was significantly reduced in ginseng-pretreated WT mice (11.47 ± 0.94% vs. 6.82 ± 0.73%; *p* < 0.01), but not in the Nrf2^−/−^ mice (14.92 ± 1.32% vs. 12.35 ± 1.03%. *p* > 0.05), suggesting the contribution of Nrf2 signals in ginseng protection. Together, these results provide direct, supported in vivo evidence of Nrf2 benefits and the Nrf2-dependent ginseng protection on long-term recovery after permanent distal cerebral ischemia.

### 3.2. Loss of Nrf2 Exacerbated the Long-Term Reactive Astrogliosis in the Peri-Infarct Cortex, Whereas Ginseng Attenuated Such Deteriorative Progression in an Nrf2-Dependent Manner

**Figure 2:**
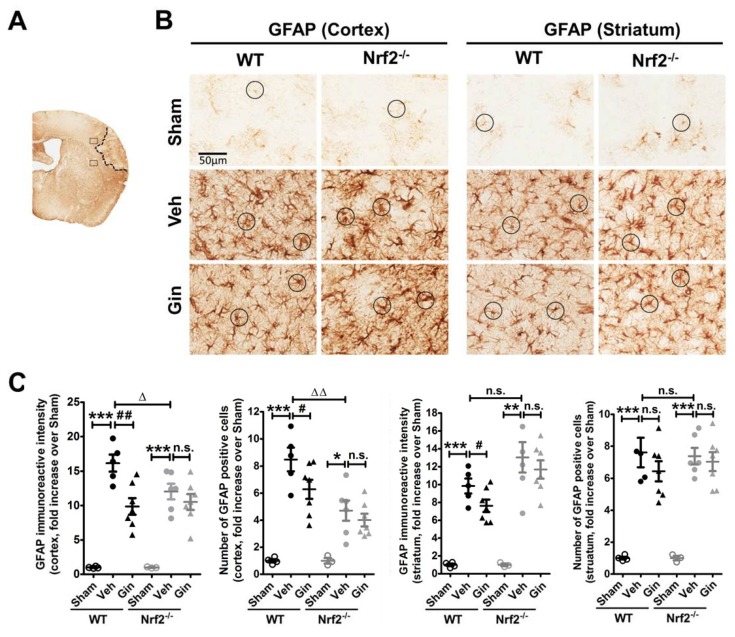
The Nrf2-associated reactive astrogliosis contributes to ginseng neuroprotection. (**A**) Schematic representation of the GFAP staining and the infarct area; the squares indicate the peri-infarct areas of the cortex and striatum for micrographic measurement. (**B**) The representative images of GFAP-positive astrocytes in the peri-infarct areas of the cortex and striatum. The round boxes indicate the typical morphology of astrocytes. (**C**) Quantitative analysis revealed the overall GFAP immunoreactive intensity and the total number of GFAP-positive cells in the cortex and striatum. In the peri-infarct cortex areas of both genotypes of mice, ischemia evoked extremely higher reactive astrogliosis, indicated by either the overall immunoreactive intensity or the total number of astrocytes, which was more severe in Nrf2^−/−^ mice. In contrast, ginseng significantly attenuated the ischemia-induced increase of reactive astrogliosis in the WT but not the Nrf2^−/−^ mice. Interestingly, no significant difference was detected in the striatum among post-pdMCAO groups, except that ginseng reduced the overall GFAP reactive intensity. * *p* < 0.05, ** *p* < 0.01, *** *p* < 0.001, ^∆^ *p* < 0.05, ^∆∆^ *p* < 0.01. *n* = 3–4 per sham group, *n* = 5–7 per ischemic group. pdMCAO: permanent distal middle cerebral artery occlusion; GFAP: glial fibrillary acidic protein; Gin: Ginseng; Veh: Vehicle; n.s.: no significance.

Ischemic insults cause pathological alteration of astrocytes, including the upregulation of GFAP, an important constituent of astrocytic intermediate filaments and hypertrophy of astrocytic processes, which can also be observed in patients who have had a stroke [34]. To examine whether astrocytes contribute to the neuroprotection of ginseng, we analyzed the morphological change of reactive astrogliosis in the peri-infarct areas of the cortex and striatum of mice at 28 days after pdMCAO, revealed by GFAP immunostaining (Figure 2). Under basal conditions, astrocytes were ubiquitously distributed in different brain regions, most of which exhibited a nonreactive state, with a small cell body and fine branches in both genotypes. In response to ischemic injury, the expression levels of astrocyte activation and proliferation were strikingly increased in both brain regions of all post-ischemia groups. This was indicated by a 10- to 15-time increase in overall GFAP immunoreactive intensity and about a 4- to 8-fold increase in the number of GFAP-positive astrocytes. Most cells displayed more highly stained somas and thick processes. 

In comparison with WT control mice, the vehicle-pretreated Nrf2^−/−^ mice exhibited a remarkably lower expression level with a number of degenerated or fragmentized astrocytes in the peri-infarct cortex area, suggesting that the absence of Nrf2 may exacerbate the ischemia-induced progression of reactive astrogliosis. Notably, no difference was detected between WT and Nrf2^−/−^ mice in the striatum area. In contrast, ginseng-pretreated WT mice, but not Nrf2^−/−^ mice, exhibited attenuated reactive astrogliosis with even distribution in both regions, indicating the contribution of the Nrf2-dependent reactive gliosis progression in the neuroprotection of ginseng. GFAP immunoreactive intensity or the number of GFAP-positive cells displayed a similar tendency. Together, consistent with our previous report [12], these findings support the hypothesis that Nrf2-associated reactive gliosis may contribute to long-term ischemic brain damage and the neuroprotection of ginseng. 

### 3.3. Nrf2 Deficiency Affected the Long-Term Expression Level of Aquaporin 4 in the Peri-Infarct Cortex, Whereas Ginseng Attenuated the Progression of This Water-Transporting Protein in an Nrf2-Dependent Manner

**Figure 3:**
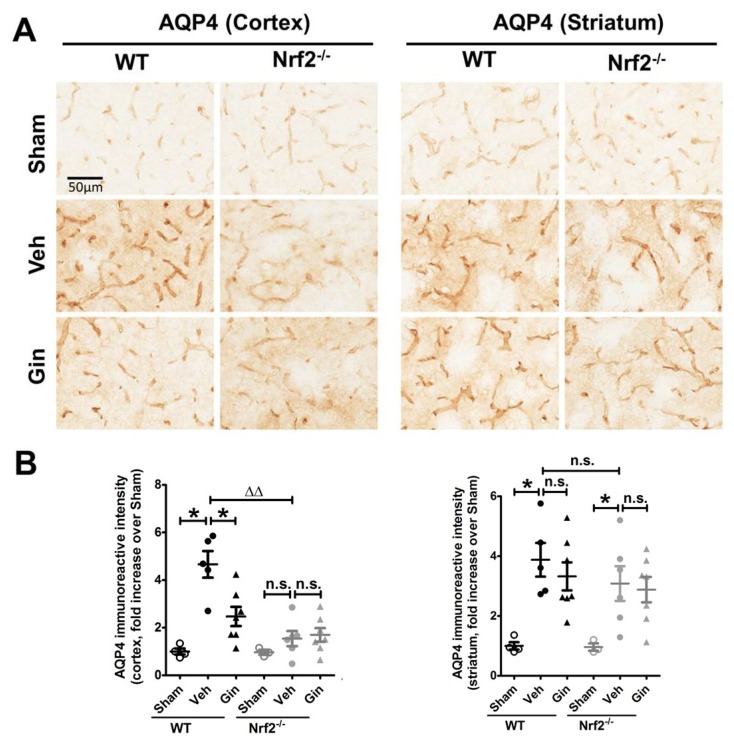
The Nrf2-associated regulation of AQP4 contributes to ginseng neuroprotection. (**A**) The representative images of AQP4 in the peri-infarct areas of cortex and striatum. (**B**) Quantitative analysis showed that ischemia injury led to a more than 5-fold increase in the cortex of WT mice, but not Nrf2^−/−^ mice. The irregular distribution pattern and some of the fragmentized AQP4 signals suggested the decline of this transmembrane water protein. In contrast, ginseng-pretreated mice displayed a markedly lower expression level of AQP4 with a regular distribution pattern. Interestingly, no significant difference was observed in the indicated striatum region among all post-pdMCAO groups. * *p* < 0.05, ^∆∆^ *p* < 0.01. *n* = 3–4 per sham group, *n* = 5–7 per ischemic group. AQP4: aquaporin 4; Gin: Ginseng; Veh: vehicle; n.s.: no significance.

Astrocytes maintain brain–water homeostasis through the transmembrane channel protein AQP4, the main member of the aquaporin family. We next measured the AQP4 expression level by immunostaining (Figure 3). At 28 days after pdMCAO, an increased expression level of AQP4 signals in both brain regions was observed in both genotypes of mice. The vehicle-pretreated Nrf2^−/−^ mice displayed significantly lower expression levels in the peri-infarct cortex area compared with vehicle-pretreated WT mice, suggesting the influence of Nrf2 on post-pdMCAO water transport. In contrast, ginseng-pretreated WT mice, but not Nrf2^−/−^ mice, exhibited attenuated reactive astrogliosis with an even distribution in the cortex region, indicating the contribution of the Nrf2-dependent reactive gliosis progression in the neuroprotection of ginseng. No significant difference was detected in the striatum area among post-pdMCAO groups. Together, the data support the hypothesis that the Nrf2-associated effect on AQP4 may be involved in long-term ischemic brain damage and the neuroprotection of ginseng.

### 3.4. Nrf2 Deficiency Exacerbated the Long-Term Ischemic Reactive Microgliosis in the Peri-Infarct Cortex and Striatum, Whereas Ginseng Alleviated such Deteriorative Progression in an Nrf2-Dependent Manner

**Figure 4:**
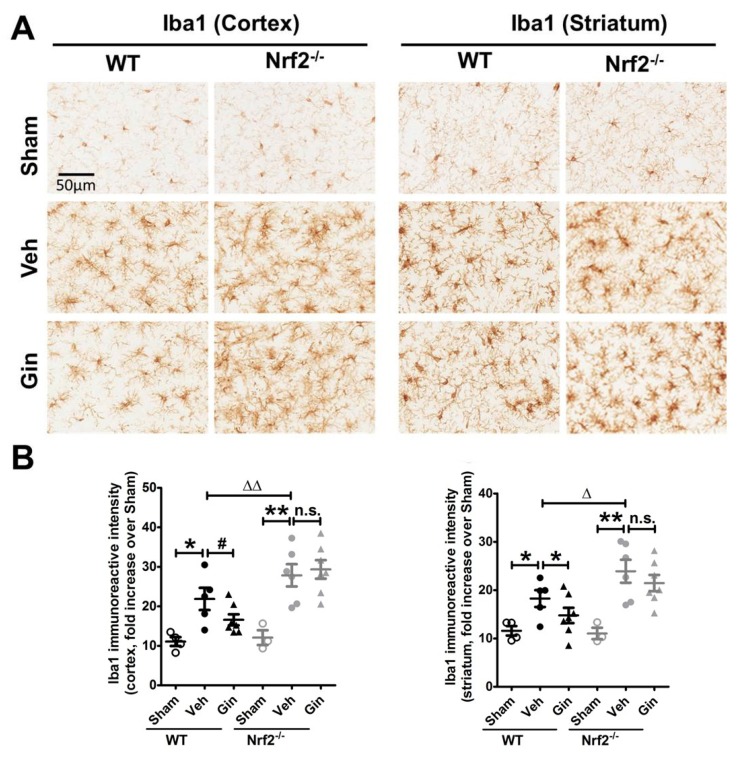
The Nrf2-associated attenuation of reactive microgliosis contributes to ginseng neuroprotection. (**A**) The representative images of Iba1-positive microglia in the peri-infarct areas of cortex and striatum, signifying the level of the reactive microgliosis. (**B**) Quantitative analysis revealed that compared with sham controls, the post-ischemia reactive microgliosis was significantly increased in the cortex and striatum regions of both genotypes, which was more severe in the Nrf2^−/−^ mice. In contrast, ginseng significantly attenuated the progression in both indicated regions of post-ischemia WT mice, but not Nrf2^−/−^ mice. * *p* < 0.05, ** *p* < 0.01, ^∆^ *p* < 0.05, ^∆∆^ *p* < 0.01. *n* = 3–4 per sham group, *n* = 5–7 per ischemic group. Iba1: ionized calcium-binding adapter protein 1; Gin: Ginseng; Veh: Vehicle; n.s.: no significance.

Meanwhile, we measured the expression level of microglia, the main cell type in ischemia-induced neuroinflammation, indicated by Iba1 immunoreactive intensity (Figure 4). At 28 days after pdMCAO, a significantly increased expression level of reactive microgliosis was observed in the peri-infarct cortex and striatum regions of both genotypes. It was more severe in post-ischemic Nrf2^−/−^ mice. In contrast, ginseng reduced the reactive microgliosis in both indicated brain regions in ischemic WT mice, but not in Nrf2^−/−^ mice. Together, these findings support the key role of Nrf2 on long-term reactive microgliosis in the pdMCAO ischemic model mice, and the attenuation of Nrf2-associated reactive microgliosis may contribute to the long-term protection of ginseng.

## 4. Discussion

In this study, we investigated the long-term effect of the loss of Nrf2 after focal permanent ischemia, and we found a significantly enlarged ischemic cortical lesion (40.4 ± 10.1%) compared with WT controls, as well as exacerbated reactive gliosis and water channel AQP4 staining, highlighting the profound salutary role of Nrf2 in long-term recovery after stroke. Interestingly, ginseng treatment conferred long-lasting neuroprotection against ischemic cortical lesion (47.3 ± 6.9%), attenuated the progression of reactive gliosis, and ameliorated the ischemia-induced AQP4 increase in WT mice after pdMCAO; however, these benefits were not observed in the absence of Nrf2, supporting the prolonged Nrf2-dependent neuroprotection of ginseng. Taken together, these findings support the critical effects of Nrf2 and the Nrf2-dependent benefit of ginseng in the pdMCAO cerebral ischemia mouse model.

### 4.1. Nrf2 in the Long-Term Recovery of Cerebral Ischemic Damage 

The long-term consequences of stroke on functional deficits and brain damage is a critical component in the translational stroke field, which needs to be better characterized [35]. To date, there is a lack of evidence regarding the functional importance of Nrf2 on long-term permanent cerebral ischemic damage. Recent studies of Nrf2^−/−^ mice and ischemic stroke have mainly focused on the acute stage of ischemic brain damage [5]. As we reported previously, Nrf2 deficiency led to remarkably larger infarct volume, not at 1 day, but at 3 days after pdMCAO, indicating the beneficial role of Nrf2 during the acute development and progression of ischemic damage [12]. This finding is supported by another group, which revealed that Nrf2 deficiency slightly enlarged the ischemic infarct lesion at 1 day, but that a significantly greater infarct size was observed at 7 days after pdMCAO [36]. The proof-of-principle experiments in this study provided direct supporting evidence of the salutary role of Nrf2 on long-term ischemic brain damage, correlated with the functional benefit in our previous report [12]. 

The Nrf2 absence led to severe reactive gliosis, which may contribute to the neuroprotection process. The Nrf2 network tightly regulates redox homeostasis and multiple intermediary metabolic processes [5]. After ischemic stress, Nr2 proteins are liberated from the Keap1 binding. The stabilized Nrf2 proteins translocate into the nucleus and activate multiple antioxidative and anti-inflammatory cytoprotective genes. The Nrf2-target genes have been reported to be preferentially activated in astrocytes, which therefore have a more efficient antioxidant defense than neurons [29]. Thus, we examined whether Nrf2 could be correlated with the long-term reactivation of astrocytes after permanent cerebral ischemia. The reactive gliosis plays a key role in modulating the fate of the connected neurons in the peri-infarct area, by modulating the synapse maintenance, cerebral blood flow, and integrity of the blood–brain barrier [22,37,38,39]. Recent studies indicate that astrocytes contribute to cell survival, angiogenesis, neuronal plasticity, and recovery days to weeks after stroke [40,41]. The inflammatory response is crucial to secondary neuronal damage after ischemia, which produces a substantial influence on both the acute damage and chronic recovery of ischemic brain tissue. Microglia, the primary resident immune cells in the brain, are constantly regulating the balance of detrimental and neuroprotective mediators that influence the fate of injured brain tissue [27,42]. 

In addition, AQP4 is the most abundant water channel protein in the brain and is specifically presented in astrocytes. As a crucial functional marker of astrocytes, AQP4 is responsible for water transport, and it plays an important role in progression and recovery following ischemic stroke (notably as part of brain edema and likely in the clearance of toxic metabolites). Thus, investigation of the AQP4 protein expression level allows us to speculate on the impact of Nrf2 on the astrocytic functional regulation of phenomena, such as water homeostasis, etc. Ischemia-induced edema is implicated in secondary brain damage. Studies indicate that the attenuation of brain edema may contribute to the recovery of cerebral ischemia [43,44]. Here, the Nrf2 absence affected the regulation of AQP4 expression, which may be associated with the alteration of edema; this is ultimately beneficial for long-term recovery [44,45,46].

### 4.2. The Nrf2-Dependent Long-Term Neuroprotection of Ginseng

Progress in understanding recovery mechanisms will ultimately promote investment in potential new approaches. Success in neuroprotection strategies could be enhanced by improving long-term outcome measures [4,47]. Recent studies over the past decade have revealed the promising potential of ginseng or ginsenosides in the stroke field; however, most reports tested only the short-term brain lesion and neurological deficits within several days of ischemic injury [48,49]. Further studies are needed to uncover the roles and mechanisms of ginseng and ginsenosides in stroke. Whether the Nrf2 mechanism plays, at least in part, an integral role in the effect of ginseng is important. This standardized ginseng extract pretreatment was shown to have sustained benefits related to ischemic damage. Such ginseng efficacy was also reported in chronic neurodegenerative disorders, such as Alzheimer’s and Parkinson’s diseases [50,51,52].

Various pieces of evidence suggest that astrocytes serve as causal or modulating factors in multiple neurological conditions [53,54]. Both glial cell types contribute to the expansion and resolution of the infarct, influencing the progression, remodeling, and repair of ischemic injury. Our previous findings revealed that the ginseng significantly attenuated the reactive gliosis progression in an Nrf2-dependent manner; such protection further enhanced long-term functional recovery in both permanent and hypoxic–ischemic stroke mouse models [12,18]. However, the long-term effects of Nrf2 function and ginseng neuroprotection on cerebral ischemic lesions remain unclear [5]. Our data showed an Nrf2-dependent manner of reactive gliosis at the late stage of ischemia, supporting the long-lasting effect of ginseng on reactive gliosis. This finding was supported by recent findings in different neurological disorders [55]. Notably, the findings of reactive gliosis were more remarkable in the surrounding cortex than in the striatum region, which correlates with the well-defined cortical infarction in the pdMCAO mouse model.

In conclusion, Nrf2 was revealed to play a critical role in the long-term recovery of permanent cerebral ischemic damage and to contribute to the neuroprotection of ginseng. The reactive gliosis and regulation of the transmembrane water channel AQP4 are implicated in this Nrf2-dependent neuroprotection.

## Supplementary Materials

Supplementary File 1
