# Supplementary material for: Nrf2 Plays an Essential Role in Long-Term Brain Damage and Neuroprotection of Korean Red Ginseng in a Permanent Cerebral Ischemia Model
Source: Antioxidants (Basel). 2019 Aug 3;8(8):273. doi: 10.3390/antiox8080273 (PMC6721128; doi:10.3390/antiox8080273)
Supplement: Supplementary File 1 [file antioxidants-08-00273-s001.pdf]

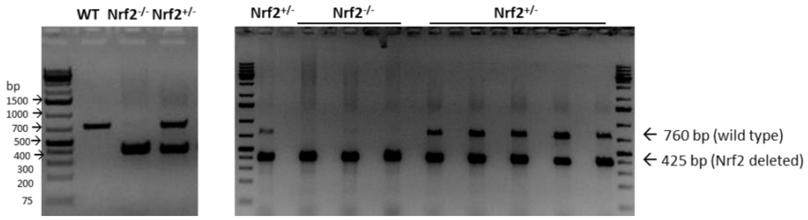

Supplementary Figure 1 The standard PCR genotyping of Nrf2$^{-/-}$ mice. Ear punch samples from Nrf2$^{-/-}$ mice were subjected to genotyping analysis. Using genomic DNA, the absence of 760 bp band and the detection of 425 bp band indicate the successful deletion of Nrf2 gene in the Nrf2$^{-/-}$ mice. Primers sequences: WT Forward, 5'-CGC CTT TTC AGT AGA TGG AGG-3'; Nrf2 Forward, 5'-GCG GAT TGA CCG TAA TGG GAT AGG-3'; Reverse, 5'-TGG ACG GGA CTA TTG AAG GCT G-3'.
